# High-Precision Depth Image Estimation for Array Gm-APD LiDAR Based on Dual-Parameter Model Feature in Dynamic Atmospheric Obscurant Environments

**DOI:** 10.3390/s26144641

**Published:** 2026-07-22

**Authors:** Yinbo Zhang, Qingyu Hou, Haoyan Wang, Boteng Zhang, Jialong Zhou, Jianfeng Sun

**Affiliations:** 1 National Key Laboratory of Laser Spatial Information, Harbin Institute of Technology, Harbin 150001, China; zhangyinbo_hit@163.com (Y.Z.); whywhy200305@163.com (H.W.); 18901798253@163.com (B.Z.); m13962878908@163.com (J.Z.); 2 Research Center for Space Optical Engineering, Harbin Institute of Technology, Harbin 150001, China; houqingyu@126.com; 3 Zhengzhou Advanced Research Institute, Harbin Institute of Technology, Zhengzhou 450000, China

**Keywords:** depth imaging, array Gm-APD LiDAR, dynamic atmospheric obscurants, dual-parameter model features

## Abstract

The nonstationary distribution of dynamic atmospheric obscurants and intense backscattering interference jointly create a severely photon-starved regime, substantially degrading the depth imaging performance of array Gm-APD LiDAR in highly scattering environments. Here, we present a depth imaging estimation algorithm through dynamic atmospheric obscurants, which enables the discrimination of atmospheric obscurant interference and significantly improves depth imaging accuracy. The proposed method employs a three-step strategy comprising data preprocessing, adaptive identification of interference-source regions, and continuous multi-frame depth image fusion based on temporal correlation, thereby enabling efficient suppression of dynamic noise and improved target integrity. The proposed method is successfully demonstrated under different attenuation lengths and dynamic atmospheric obscurant conditions. Across all tested conditions, the proposed method achieves a target recovery rate (TR) ranging from 0.71 to 0.89, a root mean square error (RMSE) ranging from 35.62 to 49.20 time bins (equivalent to 5.34–7.38 m), and a structural similarity (SSIM) ranging from 0.89 to 0.94. Compared with traditional methods, the proposed method improves TR by at least 0.41 (116.2%) and SSIM by at least 0.06 (6.8%), while reducing RMSE by at least 11.03 time bins (23.6%). In particular, under the most challenging condition, with an average attenuation length of 2.43 and an occlusion ratio of 48%, the proposed algorithm achieves a TR of 0.89, an RMSE of 49.20 time bins (equivalent to 7.38 m), and an SSIM of 0.89. These results demonstrate the considerable potential of the proposed method for depth imaging in extremely strong scattering environments.

## 1. Introduction

With the increasing maturity of single-photon detection technology, single-photon LiDAR has become an important approach for a variety of three-dimensional imaging applications because of its high optical sensitivity and low echo-energy requirement, including long-range imaging [1,2], non-line-of-sight imaging [3,4], underwater imaging [5,6,7], and imaging through scattering media [8]. Among them, Gm-APD LiDAR could detect echo signals at the single-photon level. When applied to depth imaging in atmospheric obscurant environments, it is well suited to the detection of weak echo signals under low-visibility conditions. Unfortunately, when laser photons propagate through atmospheric obscurant environments, strong backscattering from obscurant particles and background-light noise introduce increasingly severe interference. These noise photons exhibit substantial temporal overlap with the target-reflected signal photons, thereby increasing the difficulty of separating signal photons from noise photons. As a result, the scene-perception capability of Gm-APD LiDAR is severely constrained.

To mitigate the limitations of depth imaging through or within atmospheric obscurants, extensive research has been conducted on single-photon LiDAR imaging through atmospheric obscurants from three aspects: optical system optimization [9,10], advanced reconstruction algorithms [11,12,13,14], and model-fitting-based denoising. Shi [9] employs a zero-order Bessel beam with nondiffractive characteristics as the illumination beam in a single-photon LiDAR system, achieving depth imaging of a 31.5 m through an indoor smoke environment with an attenuation length of 3.8. Tobin proposes two depth imaging algorithms, M-NR3D [12] and M2R3D [13], for indoor artificial atmospheric obscurant environments. It shows that under the condition of 4.5 attenuation lengths, M2R3D can perform depth imaging of a static target 150 m away. The image resolution is 128 × 128 pixels and the signal-to-noise ratio is 0.231. Jiang [14] employs an optical transceiver system with an extremely narrow field of view and combines a 3D deconvolution algorithm based on a convex optimization solver [1] with a nonlocal neural network [15] to achieve depth imaging of a building at a range of 13.4 km in an outdoor foggy environment (2.74 attenuation lengths and a visibility of approximately 4.2 km).

Unlike the first two approaches, model-fitting-based denoising methods perform depth imaging by modeling the temporal distribution of received photons and leveraging the differences between the temporal distributions of target returns and smoke backscatter signals. Satat [16] assumes that smoke-backscattered photons and target-echo photons follow Gamma and Gaussian distributions, respectively. By estimating the two parameters of the Gamma distribution via maximum likelihood estimation, they separate target photons from scattered photons. Based on this Gamma-distribution model, they achieve depth imaging through artificial water fog in an indoor environment with a fog thickness of 57 cm and a maximum attenuation length of 2.5. Building upon the Gamma model, Liu [17] proposes a single-parameter estimation algorithm for depth imaging through smoke. It achieves depth imaging in an indoor artificial smoke environment with a smoke thickness of 1 m and an equivalent attenuation length of approximately 1.2. Peng [18] employs a Gamma model together with density-clustering-guided Gaussian model fitting to achieve depth imaging of a target at 0.7 km in an outdoor rain-fog environment with a visibility of 1.2 km. Mau [19,20] employs a mixture model (log-normal and Gaussian distributions) to fit echo signals, achieving depth imaging in an indoor artificial smoke environment. In recent years, we have utilized distribution model-fitting algorithms to achieve depth imaging through atmospheric obscurants in both indoor artificial smoke and outdoor foggy conditions [21,22,23].

However, existing algorithms for depth imaging through atmospheric obscurants have been predominantly validated under controlled and static conditions. These methods usually assume spatially uniform and complete occlusion across the field of view. In practical scenarios, interference sources may cause partial scene occlusion. This issue is particularly relevant to depth imaging through atmospheric obscurants. For array Gm-APD LiDAR, the echo signal within a single pixel may exhibit a unimodal distribution. This distribution can originate solely from the target or solely from the smoke. It may also exhibit a multimodal distribution, such as a bimodal distribution. Such a distribution may represent the target and smoke, or two distinct targets. Critically, the intensity and position of these signal peaks are nonstationary. Under such conditions, directly applying conventional algorithms or existing model-fitting methods may increase errors in target depth estimation. These methods include those proposed by Satat [16], Liu [17], Peng [18], and Mau [19,20]. The problem becomes more severe when atmospheric obscurants move rapidly. It can compromise the integrity and range accuracy of the reconstructed depth image.

To address these issues, this paper proposes a depth imaging algorithm for dynamic atmospheric obscurants based on dual-parameter model feature estimation. The algorithm mitigates interference from dynamic, localized, intense backscattering in array Gm-APD LiDAR depth imaging. The proposed approach consists of two steps. The first is a signal-level adaptive search for interference regions. The second is image-level fusion of multi-frame depth images. Dynamic atmospheric obscurants are often distributed locally within the imaging field of view. As a result, interference noise photons exhibit a non-uniform and time-varying spatiotemporal distribution. To address this problem, multi-echo separation is performed on the echo signals accumulated from all pixels. Dual-parameter model feature estimation is then used to distinguish different echo peaks. This enables adaptive identification of the temporal intervals associated with interference sources. In addition, reconstructed images from adjacent time instances exhibit strong structural similarity. This property is exploited by fusing consecutive multi-frame depth images, thereby improving the integrity of the reconstructed target image. The proposed method is therefore well suited to depth imaging with array Gm-APD LiDAR in environments containing dynamic and locally dense atmospheric obscurants.

## 2. Data Model

### 2.1. Theoretical Echo Signal Model in Atmospheric Obscurant Environments

The detection principle of LiDAR under atmospheric obscuration environments is described as follows: When the emitted laser pulse propagates through atmospheric obscuration, it is not only attenuated by suspended particles along the transmission path but also generates backscattering interactions with these particles. The backscattered photons pass through the atmospheric obscuration medium and undergo secondary attenuation before returning to the optical receiving system, where they are captured and recorded by the detector. Meanwhile, forward-scattered or unscattered photons irradiate the target surface and produce specular reflection, ultimately being received and registered by the detector. When considering background and detector noise, the echo signal expression of a single pixel of the array Gm-APD LiDAR is as follows:

(1) $$S(t)=\eta N_{f}(t)+\eta N_{t}(t)+\eta N_{b}+N_{d}$$

where *η* is the detector efficiency. *N_f_* denotes the backscattering photons. *N_t_* denotes the target reflected photons. *N_b_* represents the ambient background noise photons, which is set as a constant by default. *N_d_* represents the system noise, which is also set as a constant by default.

Since the atmospheric obscurant medium is a volume scattering medium, the backscattered echo signal can be regarded as the superposition of backscattered signals generated by scattering volume elements at different distances. Detailed theoretical derivations are provided in Appendix A. The backscattered photons of atmospheric obscurants at different distance layers are superimposed in the time domain, and the distribution expression of backscattered photons *N_f_* is obtained as follows:

(2) $$N_{f}(t)=\sum_{i=I_{start}}^{\infty }N_{R}(i)\frac{2}{\tau_{p}}\sqrt{\frac{\ln2}{\pi }}\exp\left[\frac{-4\ln2\times {\left(t-\tau_{i}\right)}^{2}}{{\tau_{p}}^{2}}\right]$$

where *τ_p_* is the laser emission pulse width. *τ_i_* denotes the flight time of reflected photons from the *i*-th scattering volume element.

Similarly, the expression for the target reflected photon *N_t_* in atmospheric obscurant environments is derived as follows:

(3) $$\left\{\begin{array}{ll} N_{t}(t)=E_{T}\eta_{T}\eta_{R}\frac{\lambda }{hc}\frac{\sigma {r_{2}}^{2}}{{\left(R_{t}/\cos\theta_{t}\right)}^{2}}\exp\left[-\alpha (R_{t}-R_{s})\frac{1+\cos\theta_{t}}{\cos\theta_{t}}\right]f(R_{t})\frac{2}{\tau_{p}}\sqrt{\frac{\ln2}{\pi }}\exp\left[\frac{-4\ln2\times {\left(t-\tau \right)}^{2}}{{\tau_{p}}^{2}}\right] & R_{s}<R_{t}<R_{s}+S_{L}\\ N_{t}(t)=E_{T}\eta_{T}\eta_{R}\frac{\lambda }{hc}\frac{\sigma {r_{2}}^{2}}{{\left(R_{t}/\cos\theta_{t}\right)}^{2}}\exp\left(-\alpha S_{L}\frac{1+\cos\theta_{t}}{\cos\theta_{t}}\right)f(R_{t})\frac{2}{\tau_{p}}\sqrt{\frac{\ln2}{\pi }}\exp\left[\frac{-4\ln2\times {\left(t-\tau \right)}^{2}}{{\tau_{p}}^{2}}\right] & R_{t}>R_{s}+S_{L} \end{array}\right.$$

In the formula, *σ* denotes the laser radar cross section (LRCS) of the target, which can be characterized by the Torrance–Sparrow model [24]. *R_t_* represents the target distance, and *τ* is the flight time of photons reflected by the target. The descriptions of other parameters in the formula are given in Appendix A.

### 2.2. Fitted Distribution Model of Echo Signal in Atmospheric Obscurant Environment

It can be seen from Equations (2) and (3) that both the time-domain echo signals of the backscattered photon *N_f_* from atmospheric obscurants and the target reflected signal photon *N_t_* contain the time-domain distribution signal *W*(*t*) of the laser transmitting pulse. In this paper, Gaussian approximation is adopted for this signal, and its distribution is given as follows:

(4) $$W(t)=\frac{2}{\tau_{p}}\sqrt{\frac{\ln2}{\pi }}\exp\left[\frac{-4\ln2{\left(t-\tau \right)}^{2}}{{\tau_{p}}^{2}}\right]$$

When the laser emission pulse passes through atmospheric obstructions such as smoke, due to the multi-path effect of particles, the backscattering echo signal will exhibit a pulse width expansion phenomenon. In this case, we use the Gamma distribution model [23] to represent the backscattering echo signal of the atmospheric obscurant. The expression of echo signal *N_f_* is as follows.

(5) $$N_{f}(t)=\delta_{f}\frac{{\beta_{f}}^{K_{f}+1}}{\Gamma (K_{f})}t^{K_{f}}\exp(-\beta_{f}t)$$

where *β_f_* is the reciprocal of the scale parameter of the smoke echo signal. *K_f_* is the shape parameter of the smoke echo signal. Γ(*K*) is the Gamma function. *δ_f_* is the intensity value of the smoke echo signal.

For the Gamma distribution model, if its shape parameter *K* is sufficiently large, the Gamma distribution approximates the Gaussian distribution. Therefore, we employ the double Gamma distribution model to represent the echo signal, and its specific form is as follows:

(6) $$S(t)=\eta \delta_{f}\frac{{\beta_{f}}^{K_{f}+1}}{\Gamma (K_{f})}t^{K_{f}}\exp(-\beta_{f}t)+\eta \delta_{t}\frac{{\beta_{t}}^{K_{t}+1}}{\Gamma (K_{t})}t^{K_{t}}\exp(-\beta_{t}t)+\eta N_{b}+N_{d}$$

where *β_t_* is the reciprocal of the scale parameter of the target echo signal. *K_t_* is the shape parameter of the target echo signal. *δ_t_* is the intensity value of the target echo signal.

For the array Gm-APD LiDAR, each photon count within a single pixel follows a Poisson distribution *P*(·), which is expressed as follows.

(7) $$H_{i}∼P\left(S_{i}\right)=\left\{\prod_{j=1}^{i-1}\exp\left(-S_{j}\right)\right\}\left\{1-\exp\left(-S_{i}\right)\right\}$$

where *H_i_* is the observed histogram distribution. Since the Gm-APD detector performs timing based on time bin, the time t in the echo signal triggering process is typically calculated in terms of bin numbers. Let *i* denote the *i*-th time bin. To compensate for the pile-up effect caused by dense atmospheric obscurants in array Gm-APD LiDAR, we use multinomial distributions to model the observed histograms. The probability *P*(*H*|*S*) of histogram *H* observed in a single pixel under atmospheric obscurant conditions is given as:

(8) $$\begin{array}{cc} P\left(H|S\right) & =\frac{N!}{H_{1}!H_{2}!\cdots H_{T}!\left(N-I^{T}H\right)!}P{\left(\mathrm{no}\mathrm{detections}\right)}^{N-I^{T}H}\prod_{i=1}^{T}P{\left(i|S\right)}^{H_{i}}\\ & =\frac{N!}{H_{1}!H_{2}!\cdots H_{T}!\left(N-I^{T}H\right)!}\exp{\left(-I^{T}S\right)}^{N-I^{T}H}\prod_{i=1}^{T}{\left\{\exp\left(-\sum_{k=1}^{i-1}S_{k}\right)-\exp\left(-\sum_{k=1}^{i}S_{k}\right)\right\}}^{H_{i}} \end{array}$$

where *T* is the maximum time bin value. ***I*** denotes a row vector of length *T* with all elements equal to 1. *S* is a column vector distribution. *N* is the total number of laser pulses. Taking the likelihood function of the above equation, we can solve for the photon count in the *i*-th time bin as follows.

(9) $$S_{i}=\ln\left(1+\frac{H_{i}}{N-\sum_{k=1}^{i}H_{k}}\right)$$

The above theoretical research establishes the correlation between the histogram data of echo signals collected by Gm-APD LiDAR and the photon distribution model of echo signals. Subsequent theoretical simulations and data processing will be completed based on the above models.

## 3. Echo Signal Feature Extraction and Processing Algorithm

### 3.1. Analysis of Echo Signal Characteristics in Atmospheric Obscurant Environment

In order to achieve discrimination between the backscattering signal and the target reflection signal in the atmospheric obstruction environment, the primary task of the research is to extract the features of the two signals. Based on the stable feature differences, the interference source can be identified and noise suppressed. In this section, based on the signal photon distribution model in Section 2.1 and the Gm-APD LiDAR trigger model, the Monte Carlo algorithm [25] will be used to simulate the echo signal in the atmospheric obstruction environment. Through numerical simulation methods, the boundary conditions of the characteristics of the backscattering signal and the target signal will be explored. The system parameters of the Gm-APD LiDAR adopted in this paper are listed in Table 1. To match the actual hardware specifications of the real experimental platform, all numerical simulation parameters in this section are consistent with those in Table 1.

**Figure 1:**
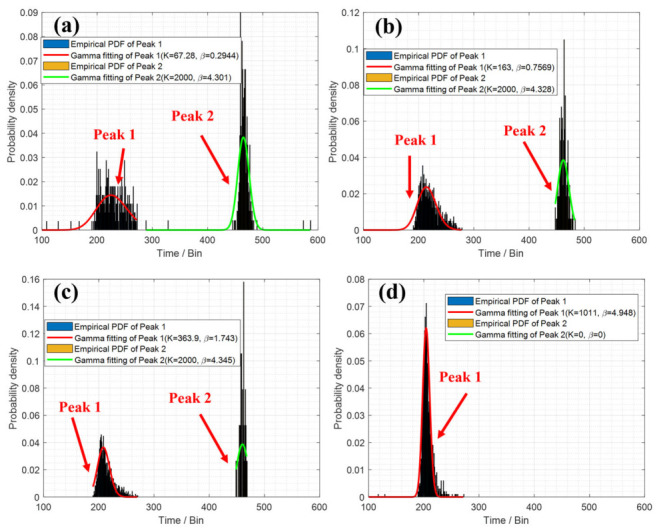
The smoke signal histograms, target signal histograms and their Gamma fitting result under different power conditions. (**a**–**d**) are the simulation results corresponding to laser powers of 10 μW, 50 μW, 100 μW, and 200 μW, respectively.

This paper conducts simulation calculations for atmospheric obscurants using dust aerosol smoke as an example. The atmospheric obscurant is distributed in the local area on the detection path between the target and the Gm-APD LiDAR. By configuring the time gate, the atmospheric obscurant is constrained within the gate of the Gm-APD LiDAR. Background light interference is neglected in the simulation. The average radius *r_m_* of dust aerosol particles [26] is 0.5 μm. Their standard deviation *σ* is 2.99 μm. At a wavelength of 1.06 μm, the complex refractive index is 1.52–8.0 × 10^−3^i. In the simulation, the smoke starts at a distance of 30 m and has a thickness of 10 m. The target is located at 70 m. The attenuation coefficient is set to 5 × 10^−4^ m^−1^. Figure 1 shows the simulated echo signal histogram. It is worth noting that the histogram heights of the smoke (Peak1) and the target (Peak2) follow an independent distribution, and the relative distribution of the histogram peak heights cannot characterize the true relative signal intensity. Under different laser power levels, the Gamma fitting parameters *K* and *β* of the target signal remain nearly constant and exhibit stable characteristics. In contrast, the fitted parameters *K* and *β* of the smoke backscattering echo signal increase gradually. This is because the triggering probability of smoke increases under high laser power, which produces an obvious pile-up effect on echo signals and ultimately leads to significant distortion of the smoke signal. When the laser power satisfies target detection requirements, the target echo signal and smoke scattering signal exhibit distinct differences in time-domain characteristics. The dual characteristic parameters obtained by Gamma fitting can realize discrimination between smoke and target signals.

**Figure 2:**
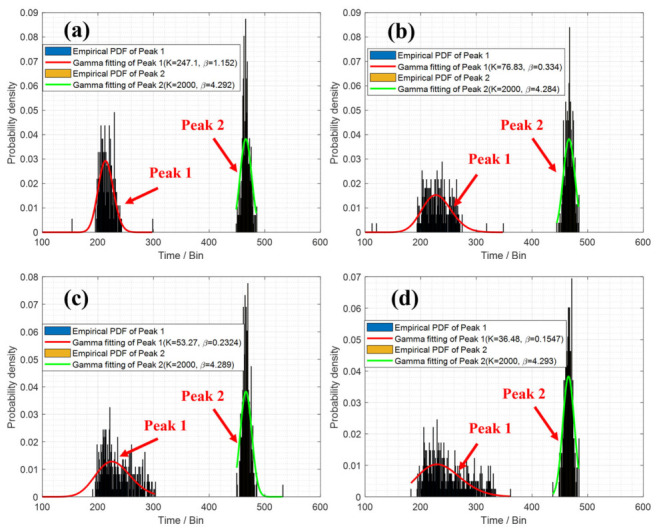
The smoke signal histograms, target signal histograms and their Gamma fitting result under different smoke thickness conditions. (**a**–**d**) are the simulation results corresponding to the thickness of the smoke is 5 m, 10 m, 15 m, and 20 m, respectively.

According to the backscattering photon distribution model, the time-domain distribution of smoke backscattering echo signals is jointly affected by the smoke thickness and laser emission pulse width. Therefore, this paper simulates the echo histograms of the Gm-APD LiDAR under different conditions of smoke thickness and laser emission pulse width. In the simulation, the smoke begins at a distance of 30 m. The laser power is 10 μW. The target is located at 70 m. The laser pulse width is 10 ns. The attenuation coefficient is set to 5 × 10^−4^ m^−1^. The simulation results of the echo signal histogram corresponding to smoke thicknesses of 5 m, 10 m, 15 m and 20 m are presented in Figure 2. With the increase in smoke thickness, the estimated parameter *K* of the smoke signal histogram decreases from 247.1 to 36.48, and the parameter *β* decreases from 1.152 to 0.1547. In contrast, the estimated parameters *K* and *β* of the target signal histogram remain almost unchanged. The results indicate that when the echo signal is fitted by the dual Gamma distribution, the variation in smoke thickness only affects the estimation results of the smoke backscattering histogram and exerts no influence on the estimation results of the target reflection signal histogram.

**Figure 3:**
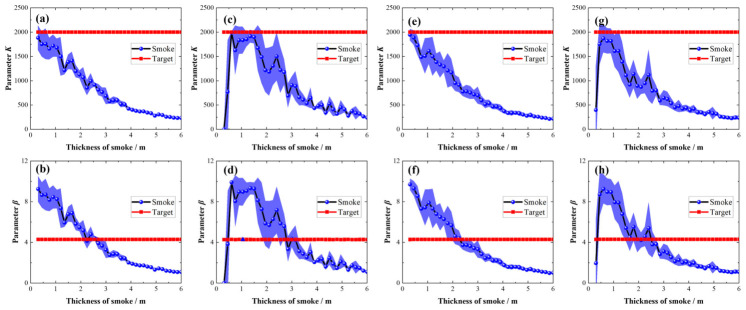
Distribution of Gamma fitting parameters of smoke and target signal histograms with variable smoke thickness. Solid lines: mean values from repeated Monte Carlo simulations under varying smoke thicknesses (red square-dotted line: target; blue circular markers: smoke). Shaded areas: standard deviation error bands around the mean. (**a**,**b**) show the distributions of the *K* and *β* parameters, respectively, for a laser power of 10 μW and an attenuation coefficient of 5 × 10^−4^ m^−1^. (**c**,**d**) show the distributions of the *K* and *β* parameters, respectively, for a laser power of 1 μW and an attenuation coefficient of 5 × 10^−4^ m^−1^. (**e**,**f**) show the distributions of the *K* and *β* parameters, respectively, for a laser power of 10 μW and an attenuation coefficient of 1 × 10^−3^ m^−1^. (**g**,**h**) show the distributions of the *K* and *β* parameters, respectively, for a laser power of 20 μW and an attenuation coefficient of 5 × 10^−3^ m^−1^.

Figure 3 presents the statistical results of parameters *K* and *β* derived from Gamma-distribution fitting of smoke and target echo histograms. The solid lines represent the mean values from repeated Monte Carlo simulations at each smoke thickness, where the red square-dotted line corresponds to the target echo and the line with blue circular markers corresponds to the smoke echo. The shaded regions denote the error range (standard deviation) around the mean values. Overall, the Gamma distribution parameters of the target echo are unaffected by variations in smoke thickness. The shape parameter *K* remains stable at approximately 2000. And the scale parameter *β* holds steady at around 4.3. Their narrow error ranges further verify that the fitting parameters of the target echo have favorable stability and repeatability. In contrast, the mean values of both *K* and *β* for the smoke echo show an overall downward trend with increasing smoke thickness, accompanied by prominent local fluctuations.

The evolution and critical characteristics of the fitted parameters for smoke echoes exhibit notable differences under varying laser powers and smoke attenuation coefficients. Figure 3a,b show that, for a laser power of 10 μW and an attenuation coefficient of 5 × 10^−4^ m^−1^, the mean shape parameter *K* of the smoke echo ranges from 235.12 to 1885.10, and the mean scale parameter *β* ranges from 1.09 to 9.25. When the smoke thickness reaches approximately 2.55 m, the mean *β* of the smoke echo decreases to 4.1, which converges to the *β* value of the target echo. As depicted in Figure 3c,d, reducing the laser power to 1 μW induces intense irregular fluctuations in the mean parameters within the thin smoke region of 0.3–1.5 m. The thickness threshold for the parameters to enter a monotonic decreasing phase shifts significantly toward higher values, and the critical thickness at which the smoke *β* converges to the target *β* increases to 3.4–3.5 m. As shown in Figure 3e,f, increasing the smoke attenuation coefficient accelerates the decay rate of the parameters with smoke thickness, thereby reducing the critical thickness of the *β* parameter. At an attenuation coefficient of 1 × 10^−3^ m^−1^, the minimum critical smoke thickness is only 2.1 m, corresponding to a one-way photon flight time of 14 ns (1.4*τ_p_*). Figure 3g,h show that, for a laser power of 20 μW and an attenuation coefficient of 5 × 10^−3^ m^−1^, the fluctuation amplitude of the parameters in the thin smoke region is enhanced, with a critical *β* thickness of approximately 2.25 m.

Regarding the dispersion of parameter estimation, all four simulation groups follow a consistent distribution pattern: the standard deviation is large in the thin smoke region and small in the thick smoke region, which aligns with the physical property that echo photon counts attenuate with increasing smoke thickness. When the laser power is 10 μW and the attenuation coefficient is 5 × 10^−4^ m^−1^, the standard deviation of parameter *K* ranges from 14.49 to 398.17, and that of parameter *β* ranges from 0.07 to 1.95. The fitting uncertainty is most pronounced under the low laser power condition (1 μW), where the peak standard deviation of parameter *K* reaches 1060.76, on the same order of magnitude as the corresponding mean value. This indicates a significant degradation in Gamma fitting stability under low photon flux conditions. The parameter dispersion of the remaining two simulation groups is relatively moderate, with better fitting reliability across the entire thickness range compared to the low-power condition.

We further analyze the error intervals at the critical thickness where smoke and target *β* parameters converge across all simulation cases. At these critical points, the mean *β* of smoke echoes is very close to that of target echoes. In some working conditions, the upper bound of the error band nearly touches the target *β* baseline. However, the fluctuation ranges of smoke *K* are completely separated from the fixed target *K* in all cases. No parameter interval overlap occurs. This result directly proves that the shape parameter *K* can stably achieve effective discrimination between smoke and target echoes. It remains reliable even in the critical region where the scale parameter *β* loses discriminative capability. Its discrimination performance is not affected by parameter estimation uncertainty. The statistical results of the four groups draw a consistent conclusion: Increasing laser power raises the critical smoke thickness at which smoke and target parameters converge. Increasing the smoke attenuation coefficient reduces this critical thickness. When the smoke thickness is no less than 1.4*τ_p_*c/2, the Gamma-distribution discrimination method based on shape parameter *K* has sufficient reliability and robustness. It can stably distinguish target signals from interference signals in complex smoke environments.

**Figure 4:**
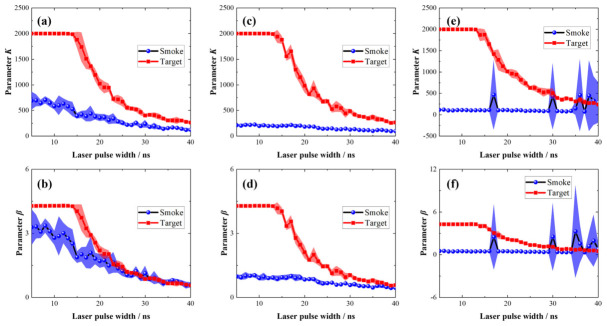
Distribution of Gamma fitting parameters of smoke and target signal histograms with different laser pulse widths. Solid lines: mean values from repeated Monte Carlo simulations under varying smoke thicknesses (red square-dotted line: target; blue circular markers: smoke). Shaded areas: standard deviation error bands around the mean. (**a**) Curve of parameter *K* distribution with smoke thickness of 3 m. (**b**) Curve of parameter *β* distribution with smoke thickness of 3 m. (**c**) Curve of parameter *K* distribution with smoke thickness of 6 m. (**d**) Curve of parameter *β* distribution with smoke thickness of 6 m. (**e**) Curve of parameter *K* distribution with smoke thickness of 9 m. (**f**) Curve of parameter *β* distribution with smoke thickness of 9 m.

To explore the boundary conditions for the influence of the laser emission pulse width *τ_p_* on the shape parameter *K*, simulation and fitting are performed on the histograms with the laser emission pulse width increasing from 5 ns to 40 ns. In the simulation, the initial smoke position is 30 m. The laser power is 10 μW. The target distance is 70 m. The laser pulse width is 10 ns. The attenuation coefficient is 5 × 10^−4^ m^−1^. The distribution results of the fitted parameters *K* and *β* under different laser emission pulse widths *τ_p_* are shown in Figure 4.

For the 3 m smoke thickness case, the fitted parameters of target echoes show obvious threshold characteristics. When the laser pulse width is no more than 12 ns, the shape parameter *K* of target echoes stays stable around 2000. The scale parameter *β* remains at approximately 4.3. The corresponding error bands are narrow. The fitting results have good repeatability and stability. When the pulse width exceeds 12 ns, both fitted parameters of smoke echoes show a continuous downward trend with increasing pulse width. The mean value of parameter *K* ranges from 124.17 to 697.09. Its corresponding standard deviation ranges from 18.79 to 191.83. The mean value of parameter *β* ranges from 0.57 to 3.32. Its standard deviation ranges from 0.08 to 0.92. In the narrow pulse region (pulse width ≤ 12 ns), the *K* parameter difference between target and smoke exceeds 1300. Their Gamma features differ significantly. This provides sufficient margin for discrimination between the two types of echoes.

When the smoke thickness increases to 6 m, the threshold evolution rule of target echo parameters is highly consistent with that of the 3 m case. Due to enhanced smoke attenuation, the overall parameter level of smoke echoes is significantly lower than that in the 3 m case. The mean value of parameter *K* ranges from 96.44 to 225.83, with a standard deviation range of 4.31 to 38.45. The mean value of parameter *β* ranges from 0.44 to 1.04, with a standard deviation range of 0.03 to 0.18. Compared with the 3 m case, the *K* parameter difference between target and smoke in the narrow pulse region is further enlarged under 6 m smoke. The dispersion of parameter estimation is lower. The consistency of Gamma fitting is better. This indicates that the narrow pulse scheme also has excellent discrimination feasibility under moderate smoke thickness.

When the smoke thickness further increases to 9 m, the mean *K* parameter of smoke echoes stays stable within 75–150 in the narrow pulse region (pulse width ≤ 12 ns). It forms a clear distinction from the stable high *K* value of target echoes. However, when the pulse width exceeds 12 ns, abnormal parameter peaks appear in smoke echoes at specific pulse width positions. This phenomenon indicates that echo photon counts are extremely low under thick smoke conditions. When the laser pulse width specifically matches the photon transit time in the smoke layer, Gamma distribution fitting suffers from severe parameter estimation bias. The dispersion degree increases sharply. This is prone to inducing erroneous discrimination between target and smoke signals. The narrow pulse region can effectively avoid such fitting instability and maintain stable discrimination performance.

In summary, under varying smoke thickness conditions, the employment of narrow-width laser emission pulses facilitates effective discrimination between smoke and target signals using the estimated shape parameter *K*. When the laser emission pulse width is no more than 12 ns, the shape parameter *K* of target echo signals presents a stable high-value distribution characteristic. It differs significantly from the low *K* value of smoke echoes. This provides a stable feature baseline for target discrimination in complex smoke environments.

### 3.2. Depth Image Estimation Method Through Atmospheric Obscurants

**Figure 5:**
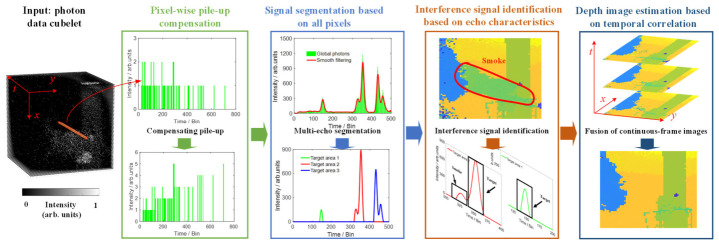
Algorithm flow for interference-source discrimination and depth image reconstruction based on dual-parameter model feature extraction.

Based on the significant feature differences in *K* and *β* estimated from the smoke and target histograms, this paper achieves classification of atmospheric obscurants and targets through dual-parameter model feature estimation of echo signals. The proposed algorithm adopts a three-step strategy, including data preprocessing, adaptive search for interference-source regions, and continuous multi-frame depth image fusion processing based on time correlation. The aim is to achieve efficient noise removal and improvement of target integrity. The algorithm flow is shown in Figure 5.

**Figure 6:**
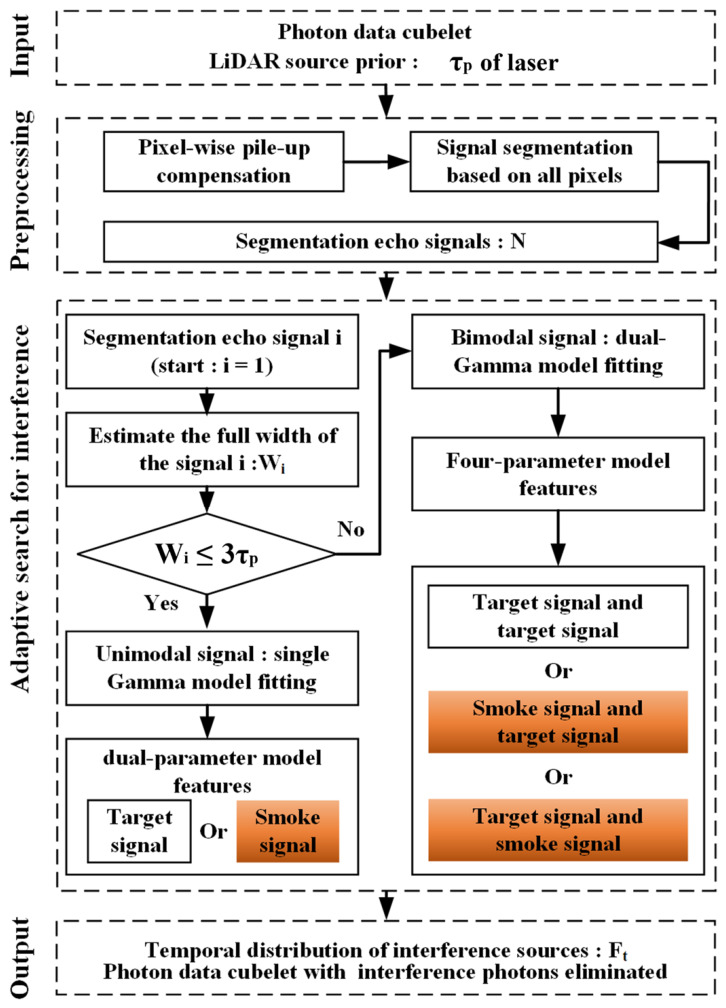
Adaptive search strategy for interference-source regions.

1. **Data preprocessing**. The array Gm-APD LiDAR data cube *H* is represented as *N_r_* × *N_c_* × *T*, where *N_r_* and *N_c_* respectively denote the number of rows and columns of the detector array. Convert *H* into *N_r_* × *N_c_* column vectors of length *T*. Use Formula (9) for pile-up compensation to obtain the vector matrix [*T*, *N_r_* × *N_c_*]. The histogram statistics result of the single pixel input in Figure 5 is the LiDAR data cube *S* (*N_r_* × *N_c_* × *T*) after pile-up compensation. Then, sum the data cube *S* to obtain a signal of length *T*. Perform threshold segmentation on the result of estimating the time profile of the full photon echo signal using the kernel density estimation method to extract multiple echo signals.
2. **Adaptive search for interference-source regions**. The segmented echo signals obtained from the previous step contain either unimodal or bimodal distributions. For unimodal signals, they may originate from either the target or smoke. For bimodal signals, they may represent combinations of smoke and target, or dual targets. To address the above signal distributions, the adaptive search strategy for interference-source regions proposed in this paper is illustrated in Figure 6. First, the characteristic parameters of unimodal or bimodal signals are calculated separately, and classification of interference sources such as target or smoke is achieved by comparing with a threshold. The threshold temporal boundary condition for separating dual-return LiDAR signals is no less than one pulse width *τ_p_* [27]. Therefore, the criterion used in this study to distinguish single-peak and double-peak signals is as follows: the full pulse width W_i_ of the separated echo signal i is calculated. If the full width Wi is no greater than 3*τ_p_*, the signal is classified as a single-peak signal; otherwise, it is classified as a double-peak signal. Through adaptive search of interference source regions, the distribution range *F_t_* of the interference source within the data range of time length *T* is determined. The photon counts within the range *F_t_* in the data cube *S* (*N_r_* × *N_c_* × *T*) are then eliminated, achieving noise removal.
3. **Continuous multi-frame depth image fusion processing**. For the echo data after eliminating interference sources, the log-matched filter algorithm [28] is used for pixel-wise signal estimation to generate depth images. The array Gm-APD LiDAR employs high-frequency laser detection technology, typically reaching kHz. The number of data frames required for single-image reconstruction is usually hundreds of Hz. The reconstruction frame rate of the depth image reaches several tens of Hz. Therefore, the adjacent reconstructed images have strong structural similarity within a short time interval. To this end, we perform histogram statistics of depth information for the reconstructed consecutive multi-frame depth images on a pixel basis. The depth image after fusion processing of consecutive multi-frame depth images is obtained through matched filtering estimation.

## 4. Experiment and Result Analysis

### 4.1. Experimental Scene and System Description

**Figure 7:**
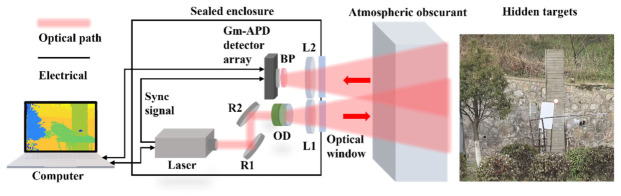
Schematic diagram of the structure and working principle of the array Gm-APD LiDAR. The optical setup includes mirrors (R1 and R2), an optical diffuser (OD), lenses (L1 and L2), bandpass filters (BP), and the Gm-APD detector array.

We evaluate the proposed algorithm using experimental data generated from outdoor artificial smoke. The array Gm-APD LiDAR is composed of 64 × 64 pixels with a central wavelength of 1064 nm. The laser pulse width is 10 ns. Each pixel is integrated with a separate TDC unit with a time bin resolution of 1 ns, corresponding to 15 cm depth resolution. Each reconstructed image is composed of multiple frames of statistical data. A schematic of the array Gm-APD LiDAR system used in these measurements is shown in Figure 7 and a summary of the key system parameters is listed in Table 1.

**Figure 8:**
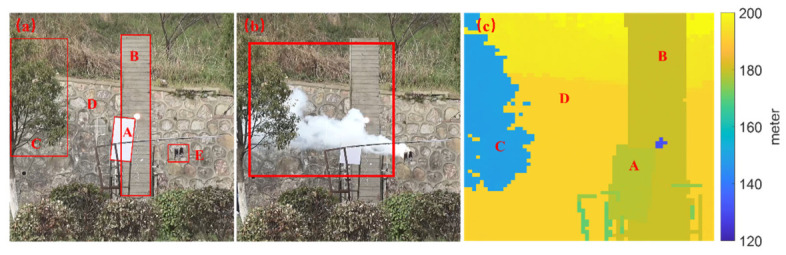
Experimental scene and ideal image result. (**a**) Scene without smoke obstruction, including white target A (178 m), wooden target B (180 m), tree C (150 m), background wall D (192 m), and hanging cage E with support (171 m). (**b**) Experimental scene with smoke obstruction; the red box corresponds to the imaging field of LiDAR. (**c**) Reference image reconstructed from multiple frame data without smoke obstruction.

To verify the adaptability of the proposed algorithm, an outdoor depth imaging through smoke experiment was conducted in this paper. The experimental scene is shown in Figure 8. The targets placed in the field of view are the white target A and the wooden target B. Their distances are 178 m and 180 m respectively. About 12 m behind target B is the background wall D, which is approximately 192 m away from the LiDAR. To achieve the obstruction of the targets A and B by the smoke, the smoke canister was placed in the hanging cage E. The cage is fixed to the front of target A by the support at a distance of 7 m. The support and the cage are about 171 m away from the LiDAR. On the left side of the field of view is tree C, with a distance of about 150 m. In the outdoor experiment, the starting position of the smoke was between tree C and the white target A. However, the atmospheric visibility on the day of the experiment is greater than 20 km. The wind speed is approximately 5–8 m/s (4-level southeast wind). During the entire experimental process, the smoke is in a non-steady-state condition. And its spatial distribution was uncertain. Achieving high-precision depth imaging in such a smoke scenario will be the core issue that the algorithm proposed in this paper needs to address.

To evaluate the depth imaging capability of the algorithm proposed in this paper through atmospheric obstructions, long-term data collection was carried out without smoke obstructions. The imaging field of view is as marked by the red box in Figure 8b. Using 60,000 frames of data for reconstruction, the scene depth image as shown in Figure 8c is obtained. The outlines of the targets A, B, C, and D are relatively clear. The support for the hanging cage E is also reflected. Subsequently, the image in Figure 8c will be used as a reference image to evaluate the image results obtained from reconstructions under different conditions.

### 4.2. Evaluation Criteria and Comparison Algorithms

Here, we use two indicators, namely the occlusion rate (*OR*) and the average attenuation length (*N_AL_*), to characterize the interference ability of atmospheric obscurant. The calculation formula for the occlusion rate is as follows.

(10) $$OR=\frac{m}{M}\%$$

where *m* represents the number of pixels obstructed by atmospheric obscurant. *M* is the total number of pixels, *M* = 4096. The criterion to calculate the occlusion rate is as follows: for the histogram echo signal within an individual detector pixel, the pixel is considered affected by smoke occlusion if the smoke echo intensity exceeds the target echo intensity. Otherwise, it is considered unaffected by smoke occlusion. The calculation of the average attenuation length *N_AL_* is obtained from the attenuation length [11] formula, as follows:

(11) $$N_{AL}=\alpha l=-\frac{1}{2}\ln\left(\sum_{i=1}^{m}\frac{n(i)}{N(i)}\right)$$

where ln is the natural logarithm. *n* is the number of returned photons in the presence of obscurant. *N* is the number of returned photons measured in the absence of obscurant. *l* is the (one-way) distance of propagation in the obscurant. *α* represents the attenuation coefficient for the level of obscurant.

We evaluate the reconstructed images using three metrics: target recovery (TR), root mean square error (RMSE), and structural similarity (SSIM) [16]. The target recovery is as follows.

(12) $$\mathrm{TR}=\frac{\sum_{i=1}^{m}m_{ef}(i)}{m}$$

(13) $$m_{ef}(i)=\left\{\begin{array}{c} 1,\;\left|d(i)-d_{s}(i)\right|\leq d_{th}\\ 0,\;\left|d(i)-d_{s}(i)\right|>d_{th} \end{array}\right.$$

where *d* is the reconstructed distance. *d_s_* is the ideal distance. *dth* is the distance error threshold. Here, half of the minimum time interval *τ_p_* for bimodal peak separation given in Ref. [27] is adopted as the boundary of the distance error threshold, corresponding to 5 time bins. *m_ef_* is the pixel that meets the range of the distance error. The root mean square error is as follows.

(14) $$\mathrm{RMSE}=\sqrt{\frac{\sum_{i=1}^{M}{\left[d(i)-d_{s}(i)\right]}^{2}}{M}}$$

It is worth noting that the RMSE values are expressed in the number of time bins. A time bin represents the minimum time interval of the detector, which is determined by the device hardware. For the array Gm-APD LiDAR adopted in this work, the time bin is 1 ns, corresponding to a photon one-way flight distance of 0.15 m.

SSIM measures image similarity from luminance (*l*), contrast (*c*) and structure (*s*).

(15) $$\mathrm{SSIM}\left(X,Y\right)=l{\left(X,Y\right)}^{\alpha }\cdot c{\left(X,Y\right)}^{\beta }\cdot s{\left(X,Y\right)}^{\gamma }$$

(16) $$l\left(X,Y\right)=\frac{2\mu_{X}\mu_{Y}+C_{1}}{\mu_{X}^{2}+\mu_{Y}^{2}+C_{1}},c\left(X,Y\right)=\frac{2\sigma_{X}\sigma_{Y}+C_{2}}{\sigma_{X}^{2}+\sigma_{Y}^{2}+C_{2}},s\left(X,Y\right)=\frac{\sigma_{XY}+C_{3}}{\sigma_{X}\sigma_{Y}+C_{3}}$$

In the formula, *α*, *β*, *γ* are the three weight parameters. *μ_X_* and *μ_Y_* represent the mean of the reference image and the defogging image, respectively, *σ_X_* and *σ_Y_* represent the variance of two images, and *σ_XY_* represents the covariance of the image. *C*_1_, *C*_2_, and *C*_3_ are constants to ensure that the denominator is not zero.

The closer the TR and SSIM are to 1, the better the algorithm’s ability to recover targets. The smaller the RMSE, the more accurate the reconstructed depth image of targets by the algorithm.

Different methods are considered for comparison as follows.

1. Peak selection algorithm (PSA) [29]: This is a classical algorithm that estimates the target distance by searching for the detection peak in the observation histogram. The target bin has the maximum number of firings and all nontarget bins have fewer than the maximum.
2. All parameter estimation algorithm (APEA) [16]: This is a probabilistic method to estimate pixel-wise fog parameters from the measurement itself without any calibration or prior knowledge. This helps to distinguish between background photons reflected from the fog and signal photons reflected from the occluded object.
3. Time gating algorithm: This is an algorithm that uses echo photon flight time segmentation to eliminate smoke noise photons. Smoke obstructions are generally located at the front end of the target. The flight time of the photons backscattered from the smoke is shorter than that of the reflected photons from the target.
4. CASPI [30]: collaborative photon processing for active single-photon imaging, a technology-agnostic, application-agnostic, and training-free photon processing pipeline for emerging high-resolution single-photon cameras. By collaboratively exploiting both local and non-local correlations in the spatio-temporal photon data cubes, CASPI estimates scene properties reliably, even under very challenging lighting conditions.

### 4.3. Results and Discussion

**Figure 9:**
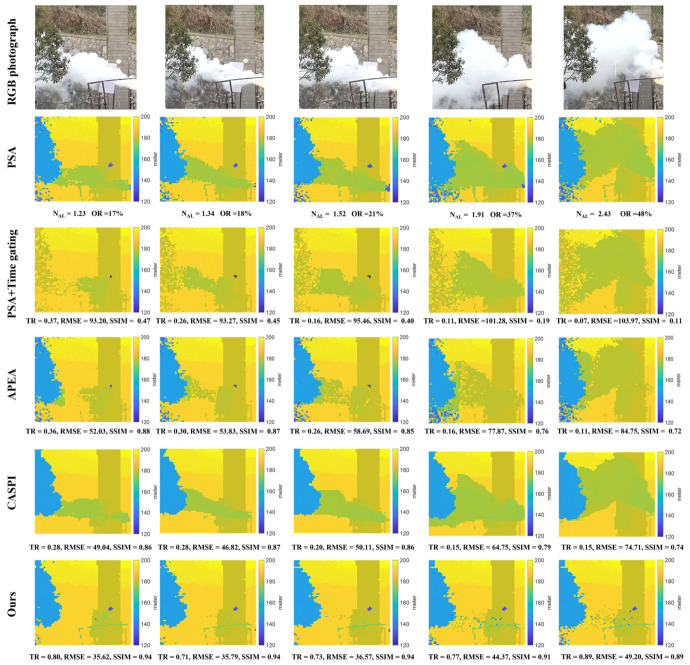
Comparison of reconstruction results under dynamic smoke obscuration interference conditions. The depth image is reconstructed by the PSA algorithm, and the occlusion rate *OR* and average attenuation length *N_AL_* are calculated based on this image. PSA + Time Gating suppresses smoke obscuration interference by using the photon flight time threshold.

Based on the experimental scenario shown in Figure 8, depth imaging of the target scene under the dynamic smoke occlusion environment was conducted. As shown in Figure 9, the top row shows the real-time RGB image of the smoke occlusion scene. The depth-reconstruction results in the subsequent rows are arranged from top to bottom as PSA, PSA + Time gating, APEA, CASPI, and the proposed method, respectively. The five columns of experimental data from left to right were obtained through continuous acquisition. The time intervals between adjacent data were within the range of 3 to 9 s.

The second row shows the depth image reconstructed by the PSA using 1000 frames of data. The depth images are similar to the RGB image of the scene. There are a large number of pixels obstructed by smoke in the reconstructed depth image. The PSA reconstructs the depth image on a pixel-by-pixel basis by locating the temporal bin with the maximum photon count in the echo histogram and using its corresponding time-of-flight as the target distance estimate. The criterion underlying PSA is consistent with that used for the OR calculation: both rely on the relative intensities of the smoke and target echoes in the raw photon-count histogram. Therefore, we use the depth image reconstructed by the PSA to calculate the occlusion rate *OR* and the average attenuation length *N_AL_*. Based on the reference image and the reconstructed depth image by the PSA, the smoke occlusion rate and the average attenuation length of five sets of experimental data are calculated. The calculation results are displayed below the reconstructed depth images of PSA. During the dynamic process from left to right, the *N_AL_*s are 1.23, 1.34, 1.52, 1.91, and 2.43, corresponding to *OR*s of 17%, 18%, 21%, 37%, and 48%, respectively. These calculation results are consistent with the changes in the RGB smoke occlusion of the scene.

Given the local distribution characteristics of smoke in the time domain, we adopt the time-separation method to suppress the smoke interference. At the same time, we combine the PSA to estimate the depth image. The depth imaging results are shown in the third row of Figure 9. By using the PSA + Time gating algorithm for noise suppression and depth imaging, partial noise removal can be achieved. However, the selection of the flight time threshold results in the loss of images of nearby trees. The TR and SSIM values of PSA + Time gating decrease from 0.37 and 0.47 to 0.07 and 0.11, respectively, while its RMSE increases from 93.20 to 103.97.

In contrast, the proposed method consistently achieves the highest TR and SSIM values and the lowest RMSE under all tested smoke conditions. Across the five conditions, its TR ranges from 0.71 to 0.89, its RMSE ranges from 35.62 to 49.20, and its SSIM ranges from 0.89 to 0.94. Compared with CASPI, the proposed method improves TR by 0.43–0.74 (153.6–493.3%) and SSIM by 0.07–0.15 (8.0–20.3%), while reducing RMSE by 11.03–25.51 (23.6–34.1%). Compared with PSA + Time gating, the proposed method improves TR by 0.43–0.82 (116.2–1171.4%) and SSIM by 0.47–0.78 (100.0–709.1%). Meanwhile, it reduces RMSE by 54.77–58.89 (52.7–61.8%).

The proposed method also outperforms APEA under all experimental conditions. Compared with APEA, it improves TR by 0.41–0.78 (122.2–709.1%) and SSIM by 0.06–0.17 (6.8–23.6%), while reducing RMSE by 16.41–35.55 (31.5–43.0%). Notably, even under the most severe smoke condition, with an *N_AL_* of 2.43 and an *OR* of 48%, the proposed method maintains a TR of 0.89, an RMSE of 49.20, and an SSIM of 0.89. These results demonstrate the superior robustness of the proposed method for depth reconstruction under different levels of smoke occlusion.

**Figure 10:**
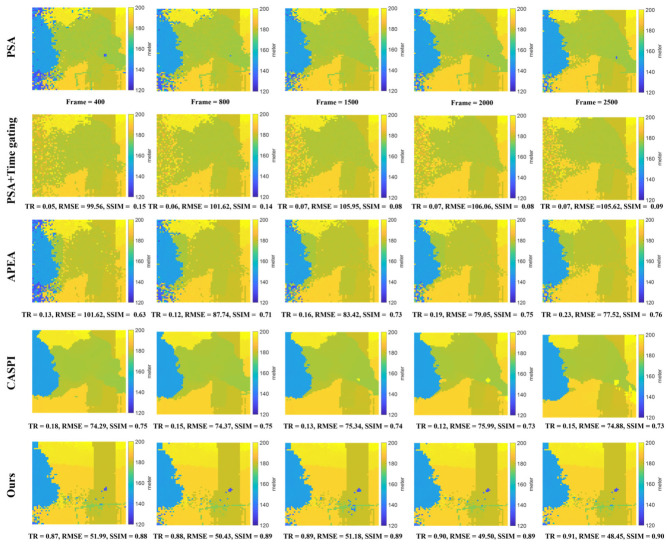
Comparison of reconstructed depth images with different numbers of data frames.

Figure 10 compares the depth-reconstruction results of different algorithms at an average attenuation length of 2.43 and a smoke-occlusion ratio of 48%, using 400, 800, 1500, 2000, and 2500 reconstructed data frames. Figure 10 presents depth images reconstructed by PSA, PSA + Time gating, APEA, CASPI and the proposed method from the first row to the last row. As the number of reconstructed frames increases, APEA shows a gradual improvement in reconstruction performance, whereas PSA + Time gating and CASPI remain substantially affected by severe smoke interference. In particular, PSA + Time gating achieves TR values of only 0.05–0.07 and SSIM values of 0.08–0.15, with RMSE values remaining high at 99.56–106.06.

In contrast, the proposed method consistently achieves the highest TR and SSIM values and the lowest RMSE under all reconstructed-frame conditions. With 400–2500 frames, its TR ranges from 0.87 to 0.91, its RMSE ranges from 48.45 to 51.99, and its SSIM ranges from 0.88 to 0.90. Compared with CASPI, the proposed method improves TR by 0.69–0.78 (383.3–650.0%) and SSIM by 0.13–0.17 (17.3–23.3%), while reducing RMSE by 22.30–26.49 (30.0–35.3%). Compared with PSA + Time gating, the proposed method improves TR by 0.82–0.84 (1171.4–1640.0%) and SSIM by 0.73–0.81 (486.7–1012.5%). Meanwhile, it reduces RMSE by 47.57–57.17 (47.8–54.1%).

The proposed method also consistently outperforms APEA. Compared with APEA, it improves TR by 0.68–0.76 (295.7–633.3%) and SSIM by 0.14–0.25 (18.4–39.7%), while reducing RMSE by 29.07–49.63 (37.4–48.8%). Notably, even with only 400 reconstructed frames, the proposed method achieves a TR of 0.87, an RMSE of 51.99, and an SSIM of 0.88. These results demonstrate that the proposed method maintains superior depth-reconstruction accuracy and structural fidelity under severe smoke occlusion, even under limited-frame conditions.

As shown in Figure 10, the proposed method maintains relatively high TR and SSIM values and a low RMSE even when the number of accumulated frames is reduced to 400. This robustness under ultra-low-photon conditions can be attributed to the complementary effects of dual-parameter feature screening and multi-frame fusion.

First, the dual-parameter feature-screening strategy jointly evaluates two complementary characteristics of the photon-return signal, rather than relying only on the amplitude or temporal position of a single echo peak. This joint criterion suppresses isolated noise photons and smoke-induced backscatter components that may exhibit partial similarity to target returns in one feature dimension. Consequently, the probability of incorrectly retaining interference photons or rejecting valid target photons is reduced, enabling more reliable identification of target-related photon events in sparse measurements.

Second, multi-frame fusion aggregates valid photon information from multiple frames while reducing the influence of randomly distributed noise and temporally fluctuating interference. Although a 400-frame measurement contains substantially fewer photons than measurements with higher frame numbers, the target-return photons preserved by the feature-screening stage can still be reinforced through fusion, whereas uncorrelated background noise and backscatter photons are effectively averaged out or excluded. Therefore, the proposed method can retain the spatial structure and depth continuity of the target under low-photon conditions.

As the number of reconstruction frames increases from 400 to 2500, the available target-photon count further increases, leading to progressively improved depth accuracy and image quality. Nevertheless, the relatively stable performance at 400 frames demonstrates that the proposed dual-parameter screening and multi-frame fusion strategy provides effective robustness for photon-starved imaging scenarios.

## 5. Conclusions

This paper establishes a detection model for Gm-APD LiDAR in atmospheric obscuration environments and conducts numerical simulations under different smoke occlusion conditions. A dual-Gamma fitting model is adopted to fit echo signal histograms, enabling the extraction of echo signal features using two parameters. The parametric boundary conditions of target and smoke echo characteristics are investigated under varying smoke thicknesses and laser pulse widths. The simulation results show that, when the smoke thickness is no less than 1.4*τ_p_c*/2, the estimated shape parameter *K* can be effectively used to discriminate between smoke and target echo signals. In addition, the shape parameter *K* of target echo signals exhibits a stable distribution when the laser pulse width is limited to within 12 ns.

Based on these characteristics, this study proposes a depth imaging algorithm for dynamic atmospheric obscurants based on dual-parameter model-feature estimation. The proposed algorithm mitigates strong backscattering interference in array Gm-APD LiDAR depth imaging through signal-level discrimination and extraction of smoke interference sources. The algorithm consists of three steps: data preprocessing, adaptive identification of interference source regions, and continuous multi-frame depth image fusion based on temporal correlation. These steps effectively suppress noise and improve target integrity. The proposed method is successfully demonstrated under various attenuation lengths and occlusion ratios. Across all tested conditions, the proposed method achieves a target recovery rate ranging from 0.71 to 0.89, a root mean square error ranging from 35.62 to 49.20 time bins (equivalent to 5.34–7.38 m), and a structural similarity ranging from 0.89 to 0.94. In particular, under the most challenging condition, with an average attenuation length of 2.43 and an occlusion ratio of 48%, the proposed algorithm achieves a TR of 0.89, an RMSE of 49.20 time bins (equivalent to 7.38 m), and an SSIM of 0.89. Compared with the best-performing benchmark method among PSA, PSA + Time gating, APEA, and CASPI, the proposed method improves TR and SSIM by 493% and 20%, respectively, and reduces RMSE by 34% under this condition. Moreover, the proposed algorithm demonstrates the capability to stably reconstruct depth images using a limited number of data frames.

This study successfully extends single-photon depth imaging through atmospheric obscurants from an indoor environment to an outdoor environment, significantly improving the imaging capability of array Gm-APD LiDAR in complex and dense smoke environments. However, the current validation is conducted only in a specific outdoor scenario involving static targets partially occluded by locally generated dynamic artificial smoke. Therefore, the present method is mainly applicable to three-dimensional imaging of static scenes under partial dynamic artificial-smoke interference. Its performance in dynamic scenes and under other types of obscurants or atmospheric conditions, such as natural fog, haze, rain, and varying aerosol densities, has not yet been systematically evaluated. In addition, the real-time capability of the algorithm, particularly its efficiency in rapidly detecting smoke within an integrated software system, requires further assessment in terms of computational time and resource consumption. Future work will focus on conducting more extensive experiments and algorithmic validation under diverse environmental conditions and dynamic scenarios. Integrating deep-learning-based detection and depth-reconstruction strategies may further improve the detection efficiency, robustness, and overall depth-reconstruction performance of the proposed method.

## Figures and Tables

**Table 1:** Summary of the main parameters of the Gm-APD LiDAR system.

System Parameters	Value
Laser wavelength (*λ*)/nm	1064
Laser repetition frequency/kHz	2.5
Laser pulse width (*τ_p_*)/ns	10
Transmitting optical system efficiency (*η_T_*)	0.95
Receiving optical system efficiency (*η_R_*)	0.95
Detector detection efficiency (*η*)	0.1
Center axis distance of transmitting and receiving optical systems (*d*_0_)/mm	50
Laser transmitted spot radius (*r*_1_)/mm	2.5
Radius of the receiving optical system (*r*_2_)/mm	35
Diameter of camera lens/mm	70
Divergence angle of laser/mrad	17.5
Divergence angle of camera lens/mrad	17.5
Dark count rate/kHz	10
BP/nm	1064 ± 1
InGaAs Gm-APD camera	64 × 64 pixels
Working wavelength/nm	900~1650
Timing jitter (camera)/ns	≤1
Bin width/ns	1

## Data Availability

The data presented in this study are available upon request from the corresponding author.

## Abbreviations

The following abbreviations are used in this manuscript:

LRCS	Laser radar cross section
OR	Occlusion rate
N_AL_	Average attenuation length
TR	Target recovery
RMSE	Root mean square error
SSIM	Structural similarity
PSA	Peak selection algorithm
APEA	All parameter estimation algorithm
CASPI	Collaborative photon processing for active single-photon imaging

## Appendix A

Since the atmospheric obscurant medium is a volume scattering medium, the backscattered echo signal can be regarded as the superposition of backscattered signals generated by scattering volume elements at different distances. In the calculation of backscattering noise photons from atmospheric obscurants, the atmospheric obscurant is approximately treated as a uniform and steady-state scattering medium, whose state does not change abruptly within a short time interval. If the thickness Δ*R* of the divided scattering volume element is sufficiently small, it can be approximated that only single scattering occurs when the laser interacts with the scattering volume element. The expression for the number of scattered photons of the *i*-th scattering volume element is given as follows:

(A1) $$N_{B}(i)=E_{T}\eta_{T}\frac{\lambda }{hc}\left\{\exp\left[-\alpha (i-1)\Delta R\right]-\exp(-\alpha i\Delta R))\right\}\frac{\beta }{\alpha }$$

In the formula, *E_T_* denotes the single-pulse emission energy of the laser. *η_T_* is the efficiency of the transmitting optical system. *λ* represents the laser wavelength. *h* is the Planck constant. *c* is the speed of light. *α* is the attenuation coefficient of the laser in the medium. *β* stands for the scattering coefficient of the medium, whose expression is given as follows [31].

(A2) $$\beta =2\pi \int_{0}^{\pi }\beta (\theta )\sin\theta d\theta$$

where *θ* is the included angle between the center of the illumination spot and the central axis of the receiving field of view. The expression of the included angle *θ_i_* corresponding to the *i*-th scattering volume element is given as follows:

(A3) $$\theta_{i}=arc\tan(\frac{d_{0}}{i\Delta R})$$

where *d*_0_ is the center distance between the transmitting and receiving fields of view of the LiDAR. The ratio of the backscattering intensity at different scattering angles to the total backscattering intensity is defined as *β*′(*θ*) = *β*(*θ*)/*β* [32]. *P*(*θ*) denotes the scattering phase function of a single spherical scattering particle [33]. After normalization of *P*(*θ*), the distribution of *β*′(*θ*) for the scattering volume element can be approximately obtained.

According to the LiDAR range equation, the calculation expression for the backscattered photons of the *i*-th scattering volume element is derived as follows:

(A4) $$\left\{\begin{array}{cc} N_{R}(i)=0 & i\Delta R<R_{s}\\ N_{R}(i)=N_{B}(i)\eta_{R}\sum_{\theta =\pi /2}^{\pi }\beta^{\prime }(\theta )\frac{\pi {r_{2}}^{2}}{\pi {\left(i\Delta R/\cos\theta_{i}\right)}^{2}}\exp\left(-\alpha \frac{i\Delta R-R_{s}}{\cos\theta_{i}}\right)f(R) & R_{s}<i\Delta R<R_{s}+S_{L}\\ N_{R}(i)=N_{B}(i)\eta_{R}\sum_{\theta =\pi /2}^{\pi }\beta^{\prime }(\theta )\frac{\pi {r_{2}}^{2}}{\pi {\left(i\Delta R/\cos\theta_{i}\right)}^{2}}\exp\left(-\frac{\alpha S_{L}}{\cos\theta_{i}}\right)f(R) & i\Delta R>R_{s}+S_{L} \end{array}\right.$$

where *η_R_* is the efficiency of the receiving optical system. *r*_2_ is the receiving radius of the LiDAR. *R_s_* denotes the starting position of the atmospheric obscurant. *S_L_* represents the thickness of the atmospheric obscurant. *f*(*R*) is the geometric overlap factor of the transmitting and receiving optical fields of view, and its expression is given as follows [34]:

(A5) $$f(R)=\left\{\begin{array}{ccc} 0 & , & d_{0}\geq r_{1}+r_{2}\\ \frac{r_{1}^{2}(\omega_{1}-\sin\omega_{1})+r_{2}^{2}(\omega_{2}-\sin\omega_{2})}{2\pi r_{1}^{2}} & , & \omega_{1}<\pi ,\;\;d_{0}<r_{1}+r_{2}\\ \frac{r_{1}^{2}(\omega_{1}+\sin\omega_{1})+r_{2}^{2}(\omega_{2}-\sin\omega_{2})}{2\pi r_{1}^{2}} & , & \omega_{1}>\pi ,\;\;d_{0}<r_{1}+r_{2} \end{array}\right.$$

where *ω*_1_ and *ω*_2_ are respectively the central angles of the overlapping region with respect to the laser spot and the cross-sectional circle of the receiving field of view. *r*_1_ denotes the radius of the laser spot.
